# PhageWeb – Web Interface for Rapid Identification and Characterization of Prophages in Bacterial Genomes

**DOI:** 10.3389/fgene.2018.00644

**Published:** 2018-12-18

**Authors:** Ailton Lopes de Sousa, Dener Maués, Amália Lobato, Edian F. Franco, Kenny Pinheiro, Fabrício Araújo, Yan Pantoja, Artur Luiz da Costa da Silva, Jefferson Morais, Rommel T. J. Ramos

**Affiliations:** ^1^Institute of Biological Sciences, Federal University of Para, Belém, Brazil; ^2^Institute of Exact and Natural Sciences, Federal University of Para, Belém, Brazil

**Keywords:** phage, prophage, clustering, web interface, web service, characterization, bacterial genome

## Abstract

This study developed a computational tool with a graphical interface and a web-service that allows the identification of phage regions through homology search and gene clustering. It uses G+C content variation evaluation and tRNA prediction sites as evidence to reinforce the presence of prophages in indeterminate regions. Also, it performs the functional characterization of the prophages regions through data integration of biological databases. The performance of PhageWeb was compared to other available tools (PHASTER, Prophinder, and PhiSpy) using Sensitivity (Sn) and Positive Predictive Value (PPV) tests. As a reference for the tests, more than 80 manually annotated genomes were used. In the PhageWeb analysis, the Sn index was 86.1% and the PPV was approximately 87%, while the second best tool presented Sn and PPV values of 83.3 and 86.5%, respectively. These numbers allowed us to observe a greater precision in the regions identified by PhageWeb while compared to other prediction tools submitted to the same tests. Additionally, PhageWeb was much faster than the other computational alternatives, decreasing the processing time to approximately one-ninth of the time required by the second best software. PhageWeb is freely available at http://computationalbiology.ufpa.br/phageweb.

## Introduction

Phages are the most abundant organisms on earth (Rohwer, 2003), inhabiting various environments and they are able to infect various bacterial species. Phages are also an important factor in bacterial evolution through horizontal gene transfer (Ochman et al., 2000) because they allow the insertion of extrinsic genetic material that can provide new characteristics to their hosts, such as antibiotic resistance, virulence factors, operons or even genomic islands (Bernheim and Sorek, 2018). These characteristics are present in cases of diphtheria (Brüssow et al., 2004), cholera (Kim et al., 2010), and food poisoning by enterohaemorrhagic *Escherichia coli* (Tozzoli et al., 2014). Moreover, phages have biotechnological applications as cloning in phage display (Winter et al., 1994), diagnosis of infections by phagotyping (Haq et al., 2012; Schofield et al., 2012), vehicles for vaccine delivery (Jafari and Abediankenari, 2015) and phage therapy as an alternative to antibiotics (Levin and Bull, 2004). Phages also play an ecological role, helping recycle nutrients, and increasing photosynthesis in the oceans (Mann et al., 2003; Sullivan et al., 2003). These organisms have two life cycles: lytic and lysogenic. During the lytic cycle, after the successful integration in the bacterial genome, phages can perform incision and excision, or remain dormant in the genome. They are called prophages. Depending on the size of the region and the success of the insertion, the prophage may remain complete and/or become cryptic (Canchaya et al., 2003; Brüssow et al., 2004) by decay, where the remains of its genetic material can provide the host genes that benefit its survival.

Prophages can be considered a cluster of phage-like genes (Zhou et al., 2011). Computational approaches, such as clustering algorithms are used to determine if these genes are close enough to each other to constitute a prophage region (Lima-Mendez et al., 2008; Zhou et al., 2011). Moreover, an important factor for the identification of prophages is the integration of the phages into specific insertion sites, such as in the bacterial genome tRNA genes (Delesalle et al., 2016). Thus, insertions in these genes indicate extrinsic genetic material, although phages do not use these sites exclusively. In addition, G+C content has been a feature used to confirm horizontal gene transfer, the presence of genomic islands and, generally, the identification of mobile genetic elements (Langille et al., 2010). In such regions, the G+C content may be quite distinct compared to the rest of the organism’s genome, and this feature is commonly used to confirm, *in silico*, the presence of horizontal gene transfer – HGT (Eng et al., 2011).

Many bacterial genomes available in public databases contain phage DNA integrated into their chromosome and phage DNA, in some cases, can make up 10–20% of the bacterial genome (Casjens, 2003). Due to the reduced cost of sequencing of complete bacterial genomes and the high costs for detection of prophages by bench methodologies (Metzker, 2010), new in silico tools for prophage detection in sequenced genomes (Lima-Mendez et al., 2008; Zhou et al., 2011; Akhter et al., 2012) and for prediction of DNA phage sequences in metagenomic data (Amgarten et al., 2018) have been developed. These computational tools generally use an approach that identifies sets of encoding protein genes according to some similarity to known phage genes. However, some of these tools present hindrances, such as the absence of a graphical interface, slow processing and a lack of a broader methodology for finding prophages in bacterial genomes (Srividhya et al., 2007).

Thus, this work presents PhageWeb, a tool to identify prophages in bacterial genomes that considers the similarity of gene sequences against a phage database, using indicators such as alteration of G+C content and, additionally, the presence of tRNA flanking the region which can be used as an evidence of insertion site (Campbell, 2003). These parameters allow analysis of each of the regions through functional characterization with fast processing.

## Materials and Methods

### Pipeline

**FIGURE 1:**
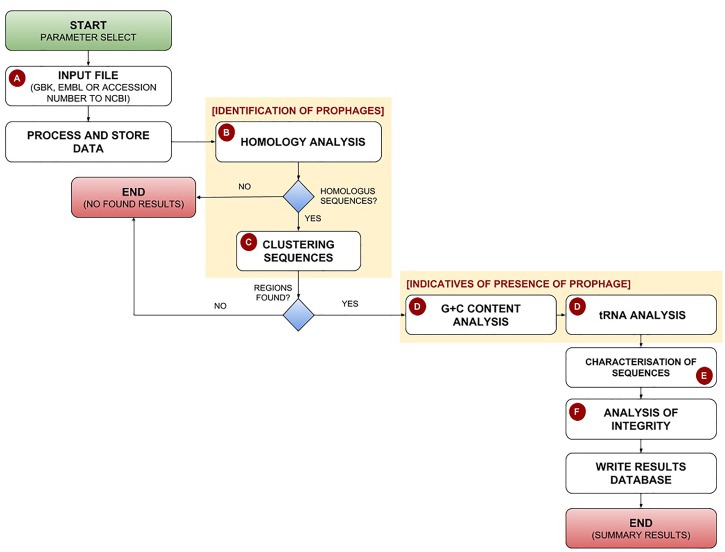
Pipeline for identification and characterization of prophages by the PhageWeb computational tool. **(A)** The pipeline receives as input the parameters (Alignment Identity and MinPts – minimum number of phage proteins in a region) and the annotation file (GenBank, Embl or the NCBI’s Accession Number) of the target genome which will be evaluated; **(B)** The homology search of the coding sequences in a local database based on the publically available sequences annotated as phage obtained from NCBI, which is automatically updated once per week; **(C)** After identifying the homology sequences, a clustering analysis based on the distance of the elements is performed: part of phage region to be evaluated on the amount of prophage there; **(D)** This optional step is useful to know if the identified region has more features which can be an evidence of prophage: G+C content (due to be possible variation of G+C content on the flanks of the prophage) and the presence of tRNAs on the flank; **(E)** Use of web services to connect biological databases to perform the functional characterization of the identified sequences; **(F)** Verification of the probable integrity of the prophage based on the composition of genes of each identified phage.

PhageWeb receives bacterial genomic sequences in GenBank or EMBL format, or the NCBI’s Accession Number of the bacterial genome as input for analysis. After, it uses the DIAMOND tool (Buchfink et al., 2015) to identify phage-homologous regions in bacterial genomes based on its own database (updated by the application itself), generating a data table that is integrated into the pipeline. The user can change the parameters to refine their analyses: MinPts (minimum number of phage proteins in a region) and the alignment identity against the phage database. Once the input data have been submitted, homology search and gene clustering step select prophage candidate regions. After G+C content and tRNA sites are identified and the characterization of the predictive sequences is performed. Finally, a phage gene conservation analysis optional is performed to indicate the possible integrity of the predicted regions, based on percentual of elements genic. If in a given region identified by PhageWeb there is an index for example of 80% or more of genes belonging to a given phage, it considers a potentially conserved region; but if the region has an index of less than 80%, it will be considered no conserved. The percentage value is optionally assigned by the user at the beginning of each analysis. The pipeline of PhageWeb is shown in Figure 1.

#### Graphical Elements

The interactive graphics for prophage regions in this application were encoded using the JavaScript component of the AngularPlasmid component^1^ – a DNA plasmid visualization component developed using Google’s AngularJS framework. AngularPlasmid provides an implementation that creates plasmid maps that are easy to use on the web. Instead of client-side JavaScript coding or other server-side programming languages, AngularPlasmid provides easy-to-use HTML markup, making generation as easy as creating a web page.

### Phage Database

The PhageWeb database consists of a collection of prophages sequences reported in several public databases. Two sources of data collection were used: the genome database of the National Center for Biotechnology Information (NCBI) database^2^ and the European Bioinformatics Institute (EBI) database^3^. The latter has an interactive environment for collecting and sharing information related to phage genomics. This way, the identified sequences were stored in a database developed in MySQL and incorporated into the application. All nucleotide sequences (FASTA and annotated files), as well as the database, are available in the tool, which is updated weekly.

#### Controlled Dataset

Eighty-four complete bacterial genomes that have predicted regions and manually annotated prophages (Casjens, 2003) were collected to be used to verify and quantify processing time, accuracy and performance of PhageWeb in relation to other software.

### Criteria for Identification of Prophage Regions

#### Clustering Algorithm

The controlled dataset (Casjens, 2003) was used to identify prophage regions by clustering known phage sequences, based on the coordinates in the genome of the homologous genes (Zhou et al., 2011). Three density-based clustering algorithms were evaluated - DBSCAN, OPTICS, and HDBSCAN – to identify the prophage candidate and to be implemented in PhageWeb. For the performance evaluation of the algorithms, four cluster evaluation metrics were used: Silhouette (Rousseeuw, 1987), Dunn (Dunn, 1974), Davies–Bouldin (Davies and Bouldin, 1979), and the Density-Based Clustering Validation index – DBCV (Moulavi et al., 2014).

#### G+C Content

To increase the precision in the identification of prophages, a method based on DNA composition (Eng et al., 2011) was used, where a sliding window of 1000 bp moves through the entire target genome to be analyzed. The sliding window divides the genome into several smaller sets (regions), and each region can be evaluated according to its G+C content (Lu and Leong, 2016). Previous studies (Eng et al., 2011) proposed the evaluation of HGT by G+C content of the genes inserted in these regions. This way, PhageWeb proposes to classify a specific region as a prophage if at least 80% of the genes show percent G+C above the mean plus one standard deviation or show percent GC below the mean minus one standard deviation.

#### Regions tRNA

Phages generally integrate into specific insertion sites. Among them, the tRNA genes of the bacterial genome (Campbell, 2003; Delesalle et al., 2016). Those sites can be used as an indication of the presence of external genetic material insertion, although phages don’t use only these places as the target for integration.

### Web Services

The functional characterization of the prophage regions is performed by integrating the results obtained in the PhageWeb identification step and public databases like UniProt, NCBI, InterPro, KEGG, Pfam and Gene Ontology through the UniProt public API by Web Service. After the integration, results can be processed and displayed in charts and tables to simplify analysis and understanding of results.

### Software

PhageWeb was developed to be a graphical interface for the rapid identification and characterization of prophages in bacterial genomes, using PHP combined with Python and Perl programming languages, besides the Bootstrap Framework. The PhageWeb tool implements an algorithm that combines similarity searches, using analysis and implementation of clustering algorithms in high density for the identification of regions in bacterial genomes. The software is available for use at: http://www.computationalbiology.ufpa.br/phageweb, and it is compatible with Mozilla Firefox 55.0.3, Opera 38.0.2 and Google Chrome 61.0. Additionally, an Application Programming Interface (API) was created to allow the external execution and, consequently, facilitating the integration of the application with other software. The API and usage instructions are available at: https://github.com/phagewebufpa/API.

### Tools Comparison

Three tools available to predict phages sequences on genomes were evaluated: Prophinder (Lima-Mendez et al., 2008), PHASTER (Arndt et al., 2016), and PhiSpy (Akhter et al., 2012).

Prophinder is one of the first web tools for prophage detection. It uses coding sequences (CDS) that are similar to those found in ACLAME database using BLAST. Based on the annotation of the ACLAME database, Prophinder selects the genes with the best correspondence to a potential prophage. PHASTER is also a web tool developed to identify phages inside bacterial genomes. Like Prophinder, it also uses homology search for prediction. PHASTER is an upgraded version of the Phast (Zhou et al., 2011) program and accepts DNA sequences data as well as annotated data in GenBank format as input. In general, PHASTER stands out for its ability to provide quality annotations with the prophage’s characteristics and to distinguish between intact and incomplete prophage. PhiSpy, however, differs from the others due to its ability to identify prophage regions that does not have any similarity to known target genes: it is not based on homology search in their predictions. PhiSpy phage detection algorithm was developed based on seven phage distinguishing characteristics: length of the protein, the direction of the transcription chain, A+T inclination and conventional G+C, the abundance of unique phage words, insertion point and similarity of phage proteins. Regarding the parameters, PHASTER, Prophinder, and PhiSpy were used with default parameter values. To compare the performance results of the computational tools, the values of Sensitivity and Positive Predictive Value will be used as evaluation metrics.

#### Sensitivity and Positive Predictive Value

The performance of PhageWeb against other platforms was evaluated using Sensitivity (Sn), representing the proportion of individuals or elements with the positive classification that yielded a positive result for a particular test, and using the Positive Predictive Value (PPV), which describes the number of true positives. Sn is obtained by: (reference prophages detected/total reference prophages) and PPV is obtained by: (reference prophages detected/(reference prophages detected + non-reference prophages detected). The alignment identity settings can be adjusted by the user of the PhageWeb, however, performance tests were based on the alignment identity set at: 80%.

## Results

### Clustering

The reference dataset had already identified and annotated prophage regions in each genome, which had several regions of prophages. With the aid of density algorithms (Zhou et al., 2011), we identified the amount of candidate according to the reference data. The algorithm that presented the best performance in the cluster identification was HDBSCAN, followed by OPTICS; the first algorithm gave the best results in the cluster evaluation metrics. For the performance evaluation of the algorithms, four cluster evaluation metrics were used: Silhouette (Rousseeuw, 1987), Dunn (Dunn, 1974), Davies–Bouldin (Davies and Bouldin, 1979), and Density-Based Clustering Validation index – DBCV (Moulavi et al., 2014). Table 1 shows the number of clusters identified by each algorithm and the average based on each of the four cluster-evaluation metrics. The HDBSCAN algorithm was selected to be used in our tool due to its best performance for identification of prophage in the genome.

**Table 1:** Performance Evaluation of Clustering algorithms in the identification of prophage regions, based on the metrics Silhouette, Dunn, Davies-Bouldin (DB), and Density-Based Clustering Validation index (DBCV).

Algorithms	Cluster	Silhouette	DBCV	Dunn	DB
Dbscan	151	0.47	-0.73323973	0.0006	0.553
Optic	168	0.54	-0.677653797	0.003	**0.51**
Hdbscan	**186**	**0.86**	**0.285253761**	**0.087**	1.2

*Silhouette – Refers to a method of interpretation and validation of data consistency within clusters; Dunn – A metric for evaluating clustering algorithms, and its purpose is to identify clusters of compact clusters, with a small variation among cluster members; Davies-Bouldin – Is a metric to validate how well the cluster was made using quantities and characteristics inherent to the data set; DBCV – This is a relative validation index for arbitrarily density-based clusters. The highlighted results (underscores) represent the algorithm mean value with the best performance in the identification and formation of clusters of the prophages according to the metrics.*

### Performance Evaluation

The comparison between PHASTER, Prophinder, PhiSpy, and PhageWeb, showed that PhageWeb was superior regarding the identification of prophages in Sensitivity (Sn) and presented positive predictive value (PPV) with the second best result compared to the other applications. For the analyzed dataset, PhageWeb reached 86.1% sensitivity and 87.3% PPV, and it is estimated that, based on the mean runtime for each analyzed genome, PhageWeb had its processing time reduced in the prediction of prophages by one-ninth of the time compared to the other tools (Table 3). The results of Sn and PPV for the dataset used can be observed in Table 2, that shows a comparison of the values.

**Table 2:** Comparative analysis of values obtained for Sn (Sensitivity) and PPV (Positive Predictive Value) between computational tools.

	Phaster	Prophinder	PhiSpy	PhageWeb
Sn	83.33%	81.02%	52.78%	86.11%
PPV	86.54%	77.43%	88.37%	87.32%

*The complete data this analysis can be observed in the Supplementary Information section.*

**Table 3:** Comparison of functionalities and features of phage prediction tools.

Resource	Phaster	Prophinder	PhiSpy	PhageWeb
Using graphical interface	Yes	Yes	No	Yes
Homology analyses	Yes	Yes	Yes	Yes
Analyses of tRNA sites	Yes	No	No	Yes
G+C content analysis	No	No	No	Yes
Results exportation	Yes	Yes	No	Yes
Circular genome view	Yes	No	No	Yes
Characterization of sequences	Yes	No	No	Yes
Alignment details	Yes	No	No	Yes
Support for biological databases integration	No	No	No	Yes
Output types	Text, graphics	Text, graphics	Text only	Text, graphics
Run time (seconds)	∼365	∼1890	∼5547	∼22

Considering the features and performance of phage identification tools, PhageWeb presents the similar features as the others, however, allowing for more complete analysis with detailing of alignment and functional characterization of the sequences: use of G+C content evidences and tRNA regions to improve the reliability of the results and shorter execution time. Runtime values were obtained experimentally from dataset bacterial genomes. A comparative analysis of the resources available for these tools can be observed in Table 3. The tests performed for the collection of this resource information were performed obeying the same standard of analysis for all the tools: same input data and only features shared by all the tools were used.

**FIGURE 2:**
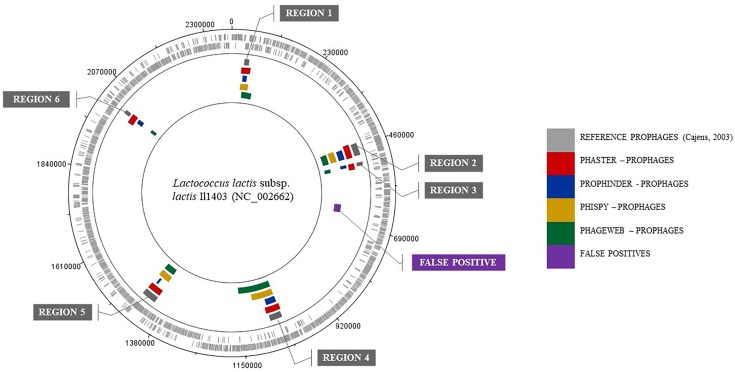
Graphical representation of the results from analyzing the genome of *Lactococcus* *lactis* subsp. *lactis* Il1403 (NC 002662) by BRIG software.

In addition, they are presented to exemplify the results obtained for a prediction of prophages for the genome of *Lactococcus lactis* subsp. *lactis* Il1403 (NC_002662). Table 4 shows the results where the coordinates (beginning and end) of the prophage regions in the reference genome are presented, along with the results from the prediction tools. The graphical representation of this analysis through software BRIG (Alikhan et al., 2011) is shown in Figure 2.

**Table 4:** Prophage regions identified by computational tools for the genome of *Lactococcus lactis* subsp. *lactis* ll1403 (NC 002662) compared to that of the lineage that was manually curated annotation.

Prophage	Reference coordinates	Phaster	Prophinder	PhiSpy	PhageWeb
Region 1	35516-49727	28461-56371	35516-49727	28818-56368	35516-72698
Region 2	447236-483244	443651-484066	451007-483244	447083-484064	447236-483552
Region 3	502723-513742	502338-520485	502723-511542	-	502723-517314
Region 4	1036642-1071558	1033815-1079175	1036642-1071558	1036482-1113152	1036642-1159446
Region 5	1414112-1456949	1414112-1457046	1439215-1446438	1415361-1457456	1415811-1456949
Region 6	2013685-2025635	1997701-2028023	2011426-2025635	-	2013685-2024681
-	False positives	-	-	633126-658623	-

## Conclusion

Despite the efficiency of existing tools for bacterial phage analysis genomes, PhageWeb presents an efficient alternative for the identification of prophages. It has high accuracy in the prediction of these organisms as well as in the evaluation of the features and simplicity of use. It also has a graphical interface that allows better interaction and flexibility to manipulate and export the resulting data. In addition, the possibility of performing other analyzes, such as GO and metabolic pathways in the same environment, simplifies the data analysis process, reducing considerably the effort applied in the interaction with biological databases.

## Data Availability Statement

The datasets analyzed for this study can be found in the PhageWeb – Dataset (http://computationalbiology.ufpa.br/phageweb/dataset/).

## Author Contributions

RR and AS conceived the idea of the program and together with DM, KP, EF, FA, and YP developed the tool computational. AL, AC, and JM evaluated the biological and computational information, defined the databases to be integrated and functions to be inserted. All authors reviewed the manuscript.

## Conflict of Interest Statement

The authors declare that the research was conducted in the absence of any commercial or financial relationships that could be construed as a potential conflict of interest.

## Footnotes

1 http://angularplasmid.vixis.com

2 http://www.ncbi.nlm.nih.gov

3 http://www.ebi.ac.uk/genomes/phage.html
