# Supplementary material for: PhageWeb – Web Interface for Rapid Identification and Characterization of Prophages in Bacterial Genomes
Source: Front Genet. 2018 Dec 18;9:644. doi: 10.3389/fgene.2018.00644 (PMC6305541; doi:10.3389/fgene.2018.00644)
Supplement: Supplementary file 1 [file Data_Sheet_1.PDF]

Supplementary Material

PhageWeb - web interface for rapid identification and characterization of prophages in bacterial genomes.

Ailton Lopes de Sousa [1], Dener Maués [2], Amália Lobato [1], Edian F. Franco[1], Kenny Pinheiro [1], Fabrício Araújo [1], Yan Pantoja [1], Artur Luiz da Costa da Silva [1], Jefferson Morais [2] and Rommel TJ Ramos. [1,*]

[1]*Federal University of Para, Institute of Biological Sciences, Belem, Para state, Brazil.*
[2]*Federal University of Para, Institute of Exact and Natural Sciences, Belem, Para state, Brazil.*

Correspondence*: Corresponding Author
rommelthiago@gmail.com

1. Supplementary Data

- Calculation of Metrics: Sensitivity and Positive Predictive Value.

Supplementary Information

Calculation of SN and PPV

Sn (Sensitivity) is obtained by: (reference prophages detected / total reference prophages)
PPV (Positive Predictive Value) is obtained by: (reference prophages detected / (reference prophages detected + non reference prophages detected).

True Positive (PV): reference prophages detected.
False Negative (FN): prophages undetected.
False Positive (FP): detected but not in the referenced prophages.

In this table are all 84 genomes used in this research as test data (Cajens, 2003). Each genome appears with its accession number in the NCBI as well as all regions (coordinate) of prophages present in its genome according to the literature. Each genome was submitted to the prophages identification software indicated in this study, including PhageWeb. The analysis response for each software based on the reference coordinates are given by the acronyms: VP, FN, FP (described above). The result count summary is displayed in the "Occurrence Count" table and the results of the Sn and PPV metrics are displayed in the "Metrics" table.

OCCURRENCES COUNT				
	PHAST	PROPHINDER	PHAGEWEB	PHISPY
TOTAL - TRUE POSITIVE (PV)	180	175	186	114
TOTAL - FALSE NEGATIVE (FN)	38	51	50	102
TOTAL - FALSE POSTIVE (FP)	28	51	27	15

METRICS				
	PHAST	PROPHINDER	PHAGEWEB	PHISPY
SN	83,33	81,02	86,11	52,78
PPV	86,54	77,43	87,32	88,37

//	GENOME	REFERENCE COORDINATES	PHASTER	PROPHINDER	PHAGEWEB	PHIPSY
1	Escherichia coli str. K-12 substr. MG1655(NC_000913)	262552- 296320	VP	FN	FN	FN
		2464567-2475651	VP	VP	VP	FN
		2754181-2775804	VP	FN	FN	FN
		564038- 584856	VP	VP	VP,VP	VP
		1410024-1432281	VP	VP	VP	VP
		1196090-1210402	VP	VP	VP	VP
		1631063-1650732	VP	VP	VP	VP
		2556793-2563354	FN	FN	FN	FN

		2064329-2076158	FN	FN	FN	FN
		**FALSE POSITIVES**	FP	--	FP	--
2	Salmonella enterica subsp. enterica serovar Typhi str. CT18 (NC_003198)	1888674-1933558	VP	VP	VP	VP
		3515470-3548975	VP	VP	VP,VP	VP
		1538899-1572919	VP	VP	VP	VP
		4473973-4507270	VP	VP	VP	FN
		1008747-1052755	VP	VP	FN	VP
		4683853-4694545	VP	VP	VP	VP
		2760475-2768771	FN	VP	FN	VP
		**FALSE POSITIVES**	FP	--	FP,FP	--
3	Streptococcus pyogenes MGAS315 (NC_004070)	1313282-1351250	VP	VP	VP	VP
		749213- 788176	VP	VP,VP	VP	VP
		1137743-1171867	VP	VP	VP	FN
		1410725-1450458	VP	VP	VP	VP
		977738-1018193	VP	VP	VP	VP
		1230304-1271815	VP	VP	VP,VP	VP
		**FALSE POSITIVES**	--	--	--	--
4	Enterococcus faecalis V583 (NC_004668)	1398097-1443843	VP	VP	VP	FN
		2817287-2829554	FN	VP	VP	FN
		2004910-2048146	VP	VP	VP	FN
		1245337-1259965	VP	VP	VP	FN
		2700666-2736808	VP	VP	VP	VP
		1923000-1962485	VP	VP	VP	FN
		289354- 326185	VP	VP	VP	VP
		**FALSE POSITIVES**	FP,FP	FP,FP,FP,FP,FP,FP,FP,FP	--	--
5	Staphylococcus	917507- 962565	VP	VP	VP	VP

	aureus subsp. aureus Mu50 (NC_002758)	2083515-2126110	VP,VP	VP	VP,VP	VP
		**FALSE POSITIVES**	FP,FP	FP,FP,FP,FP,FP,FP,FP	FP,FP	--
6	Xanthomonas axonopodis pv. citri str. 306 (NC_003919)	3105263-3115868	VP	VP	FN	VP
		1211424-1228677	FN	VP	FN	FN
		**FALSE POSITIVES**	--	FP,FP,FP,FP,FP	--	--
7	Corynebacterium diphtheriae NCTC 13129 (NC_002935)	776521- 790960	FN	FN	FN	FN
		2216802-2297805	FN	FN	FN	FN
		1866723-1880855	FN	FN	FN	FN
		154365- 190992	VP	VP	VP	FN
		**FALSE POSITIVES**	--	--	--	--
8	Streptococcus pyogenes M1 GAS (NC_002737)	527569- 571887	VP	VP	VP	VP
		1773458-1782822	VP	VP	VP	FN
		778642- 820599	VP	VP	VP	VP
		1192854-1222549	VP	VP	VP	VP
		**FALSE POSITIVES**	FP	--	FP	--
9	Staphylococcus aureus subsp. aureus N315 (NC_002745)	2005924-2049520	VP	VP	VP,VP	VP
		**FALSE POSITIVES**	FP	--	FP,FP	--
10	Lactococcus lactis subsp. Lactis Il1403 (NC_002662)	1036642-1071558	VP	VP	VP	VP
		502723- 513742	VP	VP	VP	FN
		35516- 49727	VP	VP	VP	VP
		1414112-1456949	VP	VP	VP	VP
		2013685-2025635	VP	VP	VP	FN
		447236- 483244	VP	VP	VP	VP
		**FALSE POSITIVES**	--	--	--	FP
11	Mycobacterium tuberculosis H37Rv (NC_000962)	2970551-2981576	VP	FN	VP	FN
		1780643-1788505	VP	FN	VP	FN
		**FALSE POSITIVES**	--	--	--	--
12	Neisseria meningitidis MC58 (NC_003112)	1001560-1005455	VP	FN	VP	FN
		1099910-1133980	VP	VP	VP	VP
		**FALSE POSITIVES**	--	--	--	--

13	Pseudomonas putida KT2440 (NC_002947)	4372649-4427414	VP,VP	VP , VP	VP. VP	VP
		2586633-2625819	VP	VP	VP	VP
		3418567-3447235	VP	VP	FN	VP
		1738082-1774805	VP	VP,VP	VP	VP
		**FALSE POSITIVES**	--	--	FP	--
14	Xanthomonas campestris pv. campestris str. ATCC 33913 (NC_003902.1)	2435595-2441676	VP	FN	VP	FN
		3523234-3560198	VP	FN	VP,VP	VP
		2441850-2447920	VP	FN	VP,VP	FN
		**FALSE POSITIVES**	FP	--	FP	--
15	Shigella flexneri 2a str. 301 (NC_004337.2)	2227585-2235565	FN	FN	VP,VP	FN
		238841- 251370	VP	VP	VP	FN
		899706- 918668	VP	FN	VP	VP
		311291- 328079	VP	VP	VP	VP
		2683559-2698944	VP	VP	VP	VP
		1919854-1942697	VP	VP	VP	VP
		2049694-2066397	VP	VP	VP	VP
		1175319-1188408	VP	VP	VP	VP
		1395280-1421884	VP	VP	VP	VP
		696659- 747734	VP	VP	VP,VP	VP
		**FALSE POSITIVES**	FP	--	FP,FP	--
16	Treponema denticola ATCC 35405 (NC_002967)	1169950-1202411	FN	FN	FN	VP
		**FALSE POSITIVES**	--	--	--	--
17	Clostridium acetobutylicum ATCC 824 (NC_003030)	1768546-1807515	VP	--	VP	FN
		1131796-1138899	VP	FN	VP,VP	FN
		1207416-1236260	VP	VP	VP	FN
		**FALSE POSITIVES**	--	--	FP	FP
18	Neisseria meningitidis serogroup A strain Z2491 (NC_003116)					
		10970- 22161	FN	FN	VP	FN

		1381009-1384348	FN	VP	VP	FN
		1028696-1039160	VP	VP,VP	VP	FN
		3617919-3652122	VP	VP	FN	FN
		**FALSE POSITIVES**	FP	--	--	FP,FP
19	Brucella suis 1330 (NC_004310)	578085- 584879	VP	VP	VP	VP
		**FALSE POSITIVES**	--	--	--	--
20	Escherichia coli CFT073 (NC_004431)	1169792-1228729	FN	FN	FN	FN
		909332- 942273	VP	VP	VP	VP
		3019963-3065315	VP	VP	FN	FN
		4923133-4929628	FN	FN	VP	VP
		1397370-1452231	VP	VP	VP,VP	VP
		1378215-1388952	VP	FN	VP	FN
		1327053-1372820	VP	VP	VP,VP	VP
		**FALSE POSITIVES**	FP	--	--	FP
21	Streptococcus agalactiae 2603V/R (NC_004116)	1833318-1867117	VP	VP	VP	VP
		558254- 599207	VP	VP	VP	VP
		**FALSE POSITIVES**	FP	--	FP	--
22	Pseudomonas aeruginosa PAO1 (NC_002516)	689236- 702529	VP	VP	VP	FN
		672777- 689221	VP	VP	VP	VP
		**FALSE POSITIVES**	FP	--	FP	--
23	Chromobacterium violaceum ATCC 12472 (NC_005085)	2303969-2334742	VP	VP	FN	VP
		353384- 372826	VP	VP	VP	VP
		428568- 450397	VP	VP	VP	VP
		**FALSE POSITIVES**	--	--	--	--
24	Bacillus subtilis subsp. subtilis str. 168 (NC_000964)	2151284-2285604	VP	VP,VP,VP,VP	VP	FN
		2055488-2064044	VP	FN	VP,VP	FN
		2666111-2700582	VP	VP	VP,VP	FN
		1320640-1347494	VP	VP	VP	VP
		**FALSE POSITIVES**	FP,FP	--	FP,FP	--
25	Lactobacillus johnsonii NCC 533 (NC_005362)	327894- 367791	VP	VP	FN	FN
		1292553-1330377	VP	VP	VP	FN
		**FALSE POSITIVES**	--	--	--	--

26	Clostridium perfringens str. 13 (NC_003366)	1298199-1327800	VP	VP	FN	VP
		**FALSE POSITIVES**	--	FN	--	--
27	Staphylococcus aureus subsp. aureus MW2 (NC_003923)	2046605-2088749	VP	VP	VP,VP	VP
		1529381-1573005	VP	VP	VP,VP	VP
		**FALSE POSITIVES**	FP	FP	FP	--
28	Salmonella enterica subsp. enterica serovar Typhi Ty2 (NC_004631)	4458029-4490856	VP	VP	VP	FN
		4671630-4678034	VP	FN	VP	FN
		1928058-1972330	VP	VP	VP	FN
		2735202-2754628	VP	VP	VP	VP
		1314607-1441766	VP	VP	VP	FN
		3501128-3538076	VP	VP	VP,VP	VP
		**FALSE POSITIVES**	--	--	FP	FP,FP,FP
29	Bacillus anthracis str. Ames (NC_003997)	2788307-2822836	VP	VP	VP	FN
		669361- 703908	VP	VP	FN	FN
		3075409-3126249	VP	VP,VP	VP	FN
		**FALSE POSITIVES**	--	--	--	--
30	Shewanella oneidensis MR-1 (NC_004347)	447387- 484339	VP	VP	VP	FN
		3745958-3791042	VP	VP,VP	VP	FN
		3456916-3505685	VP	VP	VP	FN
		4842149-4858240	VP	VP	VP	FN
		**FALSE POSITIVES**	--	--	FP,FP	FP,FP,FP
31	Bacillus halodurans C-125 DNA	109552 - 150419	FN	FN	VP	FN
		1028696 - 1039160	VP	VP	--	FN
		1374346 - 1384348	FN	FN	VP	FN
		3617919 - 3652122	VP	VP	VP	FN
		**FALSE POSITIVES**	--	--	FP	--
32	Bifidobacterium longum	1298692 - 1312867	VP	FN	FN	FN
		**FALSE POSITIVES**	--	--	--	--
33	Borrelia burgdorferi B31	**FALSE POSITIVES**	--	--	--	--
34	Brucella melitensis bv. 1 str. 16M	688150 - 710247	FN	FN	FN	FN
		**FALSE POSITIVES**	--	--	--	--

35	Buchnera aphidicola str. Ap	**FALSE POSITIVES**	--	--	--	--
36	Buchnera aphidicola Sg	**FALSE POSITIVES**	--	--	--	--
37	Buchnera aphidicola Bp	**FALSE POSITIVES**	--	--	--	--
38	Campylobacter jejuni NCTC 11168	**FALSE POSITIVES**	--	--	--	--
39	Chlamydia pneumoniae CWL029	**FALSE POSITIVES**	--	--	--	--
40	Caulobacter crescentus CB215	2994400-3002739	VP	--	VP	FN
		**FALSE POSITIVES**	--	--	--	--
41	Shewanella oneidensis MR-1	669361 - 705623	VP	VP	VP	VP
		2788311 - 2822840	VP	VP	VP	VP
		3075413 - 3126253	VP	VP	VP	VP
		**FALSE POSITIVES**	--	FP	--	--
42	Wigglesworthia glossinidia brevipalipis	**FALSE POSITIVES**	--	--	--	--
43	Chlamydophila pneumoniae AR39	201 - 10839	FN	FN	VP	FN
		**FALSE POSITIVES**	--	--	--	--
44	Chlorobium tepedum TLS	**FALSE POSITIVES**	--	--	--	--
45	Helicobacter pylori J99	**FALSE POSITIVES**	--	--	--	--
46	Clostridium perfringens 13	1192556 - 1294302	--	--	VP	FN
		**FALSE POSITIVES**	FP,FP	FP,FP,FP,FP	FP	FP
47	Clostridium tetani E88	189916 - 198436	FN	FN	FN	FN
		1663821 - 1696272	VP	VP	FN	VP
		2242455 - 2281387	VP	VP	FN	VP
		**FALSE POSITIVES**	FP	FP	FN	FP
48	Deinococcus radiodurans R1	71920 - 118141	FN	FN	FN	FN
		518499 - 547679	FN	FN	FN	FN
		**FALSE POSITIVES**	FP	FP,FP	--	--
49	Fusobacterium nucleatum ATCC25586	**FALSE POSITIVES**	--	--	--	--
50	Haemophilus influenzae Rd KW20	1559962 - 1594275	VP	VP	VP	VP
		1499076 - 1513073	VP	VP	VP	VP

		1636791 - 1638288	FN	FN	FN	FN
		**FALSE POSITIVES**	--	--	--	--
51	Helicobacter pylori 26695	**FALSE POSITIVES**	--	--	--	--
52	Listeria innocua CLIP1162	78505 - 114250	VP	VP	VP,VP	VP
		155931 - 166661	VP	VP	FN	FN
		1246563 - 1294235	VP	VP,VP	VP	VP
		1713958 - 1762089	VP	VP	VP	FN
		2407382 - 2445891	VP	VP,VP	VP	VP
		2591482 - 2625807	VP	VP	VP	VP
		**FALSE POSITIVES**	--	FP,FP	--	--
53	Listeria monocytogenes EGD-e	120654 - 131380	VP	VP	VP	FN
		2361897 - 2400243	VP	VP	VP	VP
		**FALSE POSITIVES**	--	--	--	--
54	Mesorhizobium loti MAFF303099	529520 - 591829	FN	FN	FN	FN
		**FALSE POSITIVES**	--	--	--	--
55	Mycobacterium leprae	**FALSE POSITIVES**	--	--	--	--
56	Mycoplasma genitalium G-37	**FALSE POSITIVES**	--	--	--	--
57	Mycoplasma penetrans HF-2	**FALSE POSITIVES**	--	--	--	--
58	Mycoplasma pneumoniae M129	**FALSE POSITIVES**	--	--	--	--
59	Oceanobacullus iheyensis HTE831	226976 - 239888	VP	VP	VP	FN
		**FALSE POSITIVES**	--	--	--	--
60	Pasteurella multocida Pm70	1426168-1466870	VP	VP	VP	FN
		**FALSE POSITIVES**	--	--	--	--
61	Ralstonia solanacearum GMI1000	879632 - 939437	VP	VP	VP	VP
		1003644 - 1018473	FN	FN	VP	FN
		1591303 - 1598986	FN	FN	FN	FN
		1781995 - 1823875	VP	VP	VP	VP
		2049291 - 2056036	FN	FN	VP	FN

		2084602 - 2127202	VP	VP	VP	VP
		2684129 - 2695788	FN	FN	VP	FN
		3486521 - 3505550	VP	FN	VP	FN
		**FALSE POSITIVES**	--	--	--	--
62	Salmonella enterica LT2	962615 - 1006520	VP	VP	VP	VP
		1098231 - 1143702	VP	VP	VP	VP
		2042047 - 2059906	FN	FN	FN	FN
		2330960 - 2345216	FN	VP	VP	VP
		2728973 - 2776825	VP	VP	VP	VP
		2844431 - 2879237	VP	VP	VP	FN
		4417931 - 4438350	VP	VP	VP	VP
		**FALSE POSITIVES**	FP	--	FP	--
63	Sinorhizobium meliloti 1021	**FALSE POSITIVES**	--	--	--	--
64	Streptococcus mutans UA159	**FALSE POSITIVES**	--	--	--	--
65	Streptococcus pneumoniae R6	**FALSE POSITIVES**	--	--	--	--
66	Streptococcus pneumoniae TIGR4	**FALSE POSITIVES**	--	--	--	--
67	Streptococcus pyogenes M18 MGAS8232	293882 - 332714	VP	VP	VP	VP
		578093 - 618765	VP	FN	VP	VP
		1041280 - 1087739	VP	VP	VP	VP
		1206360 - 1241416	VP	VP	VP	VP
		1451844 - 1493373	VP	FN	VP	VP
		**FALSE POSITIVES**	--	FP,FP	--	--
68	Streptomyces coelicolor A3(2)	6107817 - 6120760	FN	FN	FN	FN
		**FALSE POSITIVES**	FP,FP	FP	FP	--
69	Synechocystis sp. PCC 6803	**FALSE POSITIVES**	--	--	--	--
70	Thermotoga maritima MSB8	**FALSE POSITIVES**	--	--	--	--
71	Treponema pallidum Nichols	**FALSE POSITIVES**	--	--	--	--
72	Thermoanerobacter tengcongensis MB4T	**FALSE POSITIVES**	--	--	--	--
73	Vibrio cholerae N16961	1936827-1942032	FN	FN	FN	FN
		1564152-1573281	FN	FN	FN	FN
		**FALSE POSITIVES**	--	--	--	--

74	Xylella fastidiosa 9a5c	489619- 530138	VP	VP	VP	FN
		2008224-2022372	FN	VP	FN	FN
		649115- 690375	VP	VP	VP	VP
		2361812-2404268	VP	VP	VP	VP
		2177071-2183135	VP	VP	FN	FN
		1514347-1541075	VP	VP	VP	FN
		1585980-1631056	VP	VP	VP	FN
		**FALSE POSITIVES**	FP,FP	FP,FP	--	--
75	Streptococcus agalactiae NEM316	**FALSE POSITIVES**	--	--	--	--
76	Rickettsia prowazekii Madrid E	**FALSE POSITIVES**	--	--	--	--
77	Yersinia pestis CO92	964013- 971311	VP	VP	FN	FN
		1392489-1416524	FN	VP	FN	VP
		1233128-1243405	VP	FN	FN	VP
		2554373-2563203	VP	FN	FN	VP
		2363019-2413098	VP	VP	FN	VP
		2115626-2122603	VP	FN	FN	FN
		**FALSE POSITIVES**	--	--	--	--
78	Yersinia pestis KIM10	2417129-2456467	VP	FN	VP,VP	FN
		2693615-2700576	VP	VP	VP	VP
		3237524-3255252	VP	FN	FN	VP
		3389410-3399754	VP	VP	FN	VP
		3595228-3601343	FN	VP	FN	VP
		**FALSE POSITIVES**	--	FP,FP,FP,FP	FP	--
79	Xylella fastidiosa Temecula1	2005731-2020421	VP	VP	VP	FN
		1847752-1855582	FN	P	FN	FN
		1161143-1217907	VP,VP	VP,VP	VP	VP
		1117952-1142627	VP	VP	VP	FN
		454017- 467611	VP	VP	VP	VP

		1288547-1392763	VP	VP,VP	VP	VP
		1550281-1556188	FN	VP	FN	VP
		**FALSE POSITIVES**	--	FP,FP	--	--
80	Pseudomonas syringae pv. tomato str. DC3000	4515380-4530373	VP	VP	VP	FN
		625118- 647483	FN	VP	VP	FN
		2252628-2264999	VP	VP	VP	FN
		3824683-3865352	VP	VP	FN	VP
		4543843-4563495	VP	VP	VP	VP
		**FALSE POSITIVES**	FP	FP,FP,FP,FP	--	--
81	Lactobacillus plantarum WCFS1	590128- 633804	VP	VP	VP	VP
		2161732-2203820	VP	VP	VP	VP
		2998563-3007280	VP	VP	VP	VP
		2204171-2218342	VP	VP	VP	FN
		**FALSE POSITIVES**	--	FP,FP,FP	--	--
82	Bacillus cereus ATCC 14579	1805567-1866036	VP	VP	VP	VP
		2531805-2565093	VP	FN	VP	VP
		2355784-2369311	VP	VP	VP	FN
		3660466-3668825	VP	VP	VP	VP
		**FALSE POSITIVES**	FP	FP	FP	--
83	Wolbachia endosymbiont of Drosophila melanogaster	567453- 582561	VP	VP	VP	FN
		245829- 275225	VP	VP	VP	VP
		551654- 558625	VP	VP	VP	FN
		623343- 634537	VP	VP	VP	VP
		**FALSE POSITIVES**	--	--	--	FP,FP
84	Streptococcus pyogenes SSI-1	1107957-1147543	VP	VP	VP	VP
		725017- 759141	VP	VP	VP	FN
		446427- 486160	VP	VP	VP	VP
		625070- 666334	VP	VP	VP	VP
		545636- 583603	VP	VP	VP	VP
		878690- 919064	VP	VP	VP	VP
		**FALSE POSITIVES**	--	FP	--	--
